# Chronic Atypical Depression as an Early Feature of Pituitary Adenoma: A Case Report and Literature Review

**DOI:** 10.1155/2019/4892183

**Published:** 2019-07-21

**Authors:** Filipa Cardoso, Heela Azizi, Alexander Kilpatrick, Olaniyi Olayinka, Tasmia Khan, Alexa Kahn, Cecilia Canale, Chiedozie Ojimba, Olusegun Popoola, Deepa Nuthalapati, Maleeha Ahmad, Mirna Iskander, Ali Chohan, Sara Parisi, Ulunma Umesi, Hashem Kalbouneh, Arka Bhattacharya, Kodjovi Kodjo, Oluwole Jegede, Ayodeji Jolayemi

**Affiliations:** ^1^St. Matthew's University School of Medicine, Department of Psychiatry, Interfaith Medical Center, Brooklyn, New York, USA; ^2^American University of Antigua College of Medicine, Department of Psychiatry, Interfaith Medical Center, Brooklyn, New York, USA; ^3^Department of Psychiatry, Interfaith Medical Center, Brooklyn, New York, USA; ^4^Medical University of the Americas, Department of Psychiatry, Interfaith Medical Center, Brooklyn, New York, USA; ^5^Saba University School of Medicine, Department of Psychiatry, Interfaith Medical Center, Brooklyn, New York, USA

## Abstract

Pituitary adenomas are often diagnosed as incidental findings on brain imaging. We present the case of a 52-year-old African American female patient with long standing depressed mood prior to the incidental finding of a pituitary adenoma. We explore the possibility of certain mood symptoms prompting an early diagnosis of pituitary adenoma.

## 1. Introduction

Pituitary adenomas are benign clonal neoplasms derived from neuroendocrine epithelial cells of the adenohypophysis and estimated to represent approximately 25% of all clinically manifested intracranial neoplasms [1]. Pituitary adenomas share cellular characteristics with other adenomas of endocrine glands. They also often express both markers of neurosecretory granules (synaptophysin and chromogranin) as well as epithelial differentiation (cytokeratins) [1]. Dysregulation of the hypothalamic-pituitary-adrenal (HPA) axis may be potentiated by pituitary adenomas and result in the hyper- or hyposecretion of growth hormone (GH) and adrenocorticotropic hormone (ACTH) [2]. Pituitary adenomas can also be locally invasive and cause intracranial lesions that do not secrete excess hormone or yield nonspecific symptoms which can delay accurate diagnosis. Pituitary adenomas may also be asymptomatic and only be recognized as an incidental finding [2]. Early diagnosis aims to identify a cluster of neurological and endocrine symptoms; however, the literature reveals that a significant third of tumors are missed, with 22% detected incidentally on MRI scans of the brain and 14% found at autopsy [1]. A case could be made for the consideration of additional symptoms in early diagnosis of pituitary adenomas.

Neuropsychiatric symptomatology could be supplemented in the screening, detection, and early diagnosis of pituitary adenomas. The association between mood symptoms and pituitary gland hormone secretions was first highlighted in observations of abnormalities of cortisol levels in patients with depression in the late 1950s by Board et al. [3] and later in 1962 by Gibbons et al. [4]. Subsequent studies have further solidified these observations and helped create a model of mood disorders due to dysregulation of the HPA axis. In this model, individuals suffering from severe mood disorders with HPA axis hyperactivity, as manifested by hypersecretion of CRH, expressed increased cortisol levels in plasma, urine, and cerebrospinal fluid, and exaggerated cortisol responses to ACTH [5]. Anxiety, decreased concentration, fatigue, and depression have been shown to be symptoms of an increased ACTH level [6]. These results corroborate findings of a melancholic subtype of depression, while a downregulated HPA axis and CRH deficiency is more aligned with atypical depression [7]. A study by Kreitschmann-Andermahr et al. showed mild depression and weight gain in patients suffering from ACTH and GH deficiency, respectively, with depression being assessed via the Beck depression inventory [8]. These findings suggest that any disruption of the HPA axis, whether it be hyper- or hypofunctioning, could greatly impact an individual's psychological state. Such hormonal dysregulation may occur in some pituitary tumors and lead to mood symptoms at any point during the course of illness.

We present a patient who manifested with recurrent headaches and depressive symptoms prior to the emergence of other features of endocrine dysfunction due to a pituitary adenoma. A literature review was conducted to explore cases of depressive symptoms preceding the diagnosis of a pituitary adenoma. The implications for future research on the supplementation of depressive symptoms and neurological symptoms in screening tools for early diagnosis of pituitary adenomas are discussed.

## 2. Case Presentation

The patient reported is a 52-year-old African American female admitted on inpatient service for an acute depressive episode. She presented with extreme apathy, poor sleep, poor appetite, poor concentration, depressed mood, and low energy that worsened over a three-week period. The patient expressed substantial memory impairment making it difficult to establish precise details of her medical and psychiatric history. No psychotic symptoms or suicidal or homicidal ideations were reported.

Her symptoms began approximately at the age of 25 alongside a chronic course of apathy, low energy, depressed mood, interpersonal rejection sensitivity, poor self-esteem, crying spells, and hopelessness. Prior to the onset of these symptoms, she recalled frequent headaches since her teenage years which persisted during this time period. Routine evaluation at the time did not reveal any underlying organic cause and she was given a diagnosis of Major Depressive Disorder. The patient sought medical treatment at the age of 30 after experiencing dizziness, amenorrhea, and visual disturbances for one year in addition to her symptoms of depressed mood. The patient also expressed impaired memory, leaden paralysis, increased appetite, and hypersomnia. Magnetic resonance imaging (MRI) conducted at this time revealed a benign macropituitary adenoma. The patient was initially managed conservatively with hormone replacement therapy Estradiol/Progesterone 1 mg/100 mg and serial MRIs throughout the following decade. The hormone replacement therapy had little effect on her mood or cognitive dysfunction as she continued to demonstrate a depressed mood and poor cognitive functioning. The patient eventually underwent a partial transsphenoidal hypophysectomy after the pituitary adenoma of 19 mm was found extending into and compressing the optic chiasma causing visual disturbances and headaches. Following the surgery, her headaches and visual symptoms improved significantly; however, her depression and cognitive symptoms persisted. 

**Figure 1:**
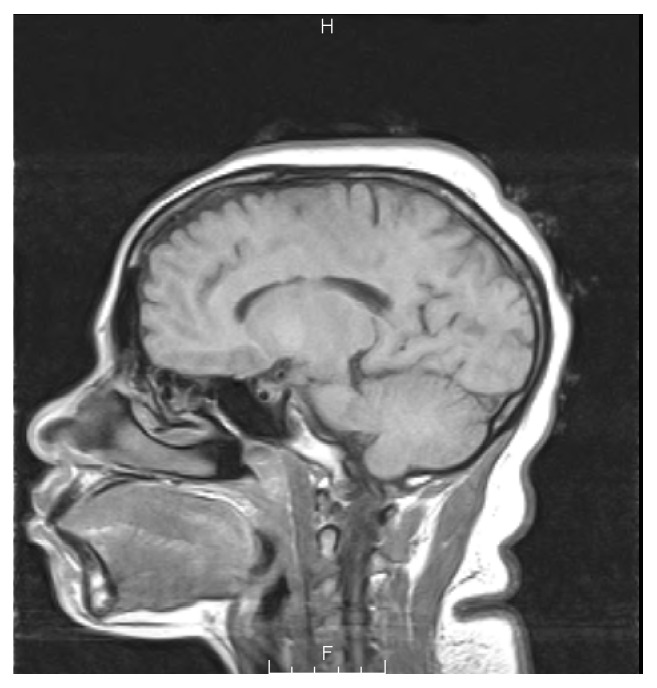
Sagittal T1 diffusion-weighted MRI of the pituitary gland.

**Figure 2:**
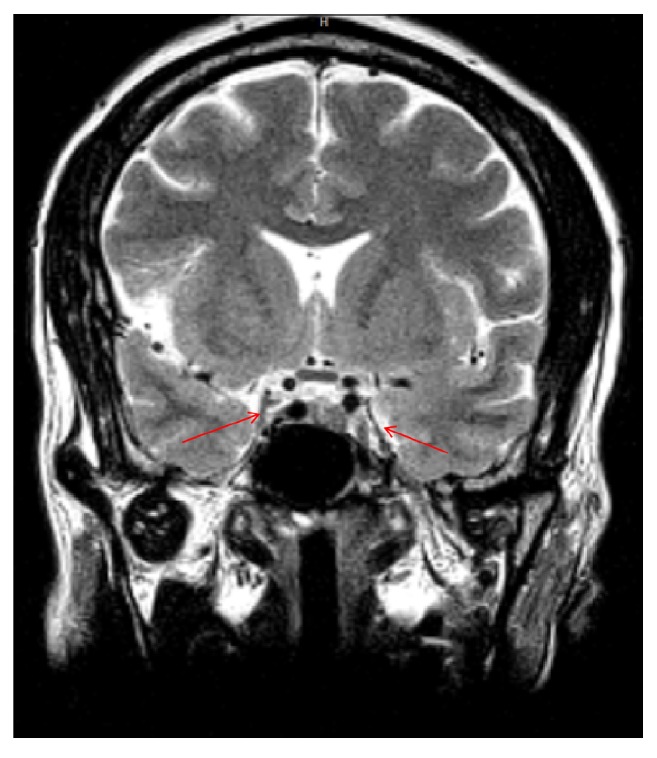
Coronal T1 diffusion-weighted MRI of the pituitary gland.

During her recent admission, we performed laboratory tests and an MRI during her current admission to further evaluate the patient (see Figures 1 and 2). Laboratory analysis is revealed in Table 1.

These values indicate panhypopituitarism with diffusely decreased levels of major endocrine hormones. FSH and LH play a key role in reproductive health and should be elevated in a postmenopausal female due to lack of estrogen inhibition. Cortisol is released from the adrenal gland in response to ACTH secretion from the pituitary gland; however, our patient demonstrated significantly decreased levels of both ACTH and cortisol levels taken in the morning at 6 am and in the evening at 4 pm. GH is the only hormone that was found to be within normal limits, although it is important to note that factors such as stress and sleep can greatly affect GH levels. Medical records obtained from previous psychiatric admissions revealed a decline in FSH and LH in the past 5 years. Neurology and endocrinology consultations both recommended no further intervention. The patient's depression was managed with Zoloft 50 mg daily for three weeks with subsequent improvement in depressed mood, hopelessness, and hypersomnia. Her interpersonal rejection sensitivity, apathy, and memory impairment for remote aspects persisted despite treatment.

## 3. Discussion

After medical work-up and psychiatric evaluation, the patient met the criteria for a depressive episode due to a disruption of the HPA axis secondary to a pituitary adenoma. She expressed mood symptoms with recurrent headaches since the age of 25, while no other indicators suggested the presence of an underlying pituitary adenoma. As a result, she was managed as a patient with Major Depressive Disorder during her early adulthood. One may still consider, however, the presence of underlying mild dysfunction of the HPA axis due to early stages of pituitary adenoma predating her psychiatric symptomatology at the age of 25 years. Its significance may have been missed by early work up until it became more severe. However this could not be corroborated from the patient's records. The diagnosis of pituitary adenoma was only made when the patient was 33 years old and presented with symptoms consistent with pituitary dysfunction, such as amenorrhea and visual disturbances. It was at this time that management of her underlying pituitary adenoma occurred. As the depressive symptoms and recurrent headaches were the only antecedents of a diagnosis of pituitary adenoma, it would be of interest to explore how depression alongside neurological symptoms such as headaches correlates with the subsequent diagnosis of pituitary adenoma. Such a correlation could provide additional opportunities for early diagnosis of pituitary adenoma, in addition to current methods used. Of note, her depressed mood improved with antidepressants during her admission. Prior attempts to treat her depression and cognitive symptoms on hormonal therapy were not successful. This is consistent with Pichot et al. [9] who proposed Pichot et al. serotonin neuroplasticity due to hormonal imbalance as an additional factor in the emergence of depression and cognitive symptoms. Hence treatment of the mood symptoms may respond not serotonergic antidepressants as opposed to hormone replacement.

We conducted a literature review to explore the correlation between depressive symptoms with neurological symptoms and subsequent diagnosis of pituitary adenoma. The literature review was a narrative targeted literature review. The review was conducted using PubMed and Google Scholar. Keywords of “pituitary lesions,” “hypopituitarism,” “mood symptoms,” “depression,” and “atypical depression” were used to find articles on the databases. Language and timeframe were not restricted due to the paucity of articles on this topic. Peer-reviewed experimental, cohort, meta-analysis, case-control, case series, and case reports that reported psychiatric symptoms and had evidence of pituitary pathology were reviewed by five authors. Further articles were added by reviewing the reference lists of included articles. Articles that were included focused on the symptomatology of pituitary injury and subsequent behavior, leading to 21 included articles that ranged in date from 1994 to 2018. Any disagreement regarding the eligibility of an article was resolved by discussion among the authors. 

Relevant data including gender, age, initial psychiatric presentation, type of depression, and lab and radiologic findings were included when available and are seen in Table 2.

Patients with hypopituitarism and depression ranged in age from 14 to 80. Of the 81% of articles reporting ages, the mean age of patients was 47 with a distribution of 57% male and 43% female. This mean age is similar to that of the patient in our case presentation at 53 years of age. The average age that patients presented with symptoms of hypopituitarism was about 33. The average age of objective findings such as findings on imaging was 47. This finding differed significantly from our patient who had an MRI conducted when hypopituitarism symptomatology started manifesting at the age of 30.

In exploring the symptoms that may predate the objective findings, symptoms of depression were reported as a psychiatric symptom in 71% of the papers. These symptoms of depression predated the pituitary findings in 51% of cases, with the average time to objective pituitary findings being 11 years from the time symptoms of depression were noted. In addition, these symptoms of mood disorder occurred concurrently with pituitary symptoms of amenorrhea, visual disturbances, sexual dysfunction, and unusual hair growth in 44% of cases, similar to the findings in our presented patient. In 41% of cases, matching up with our patient, the mood symptoms predated the pituitary symptoms of amenorrhea, visual disturbances, sexual dysfunction, and unusual hair growth. In 15% of cases symptoms of mood occurred after the onset of pituitary symptoms.

Regarding mood symptoms, 46% of the symptoms were consistent with atypical depression and 20% with melancholic depression. Several other psychiatric and neurological symptoms were reported within the papers. The most commonly reported symptoms being memory issues (29%), fatigue (24%), malaise (23%), apathy (22%), anxiety (22%), headaches (14%), sleep disturbances (14%), decreased concentration (14%), and decreased appetite (14%). Our patient presented with atypical depression, congruent with 46% of the findings; the most commonly reported symptoms were also found in our patient, such as memory issues, fatigue, malaise, and apathy. With reference to the earliest symptoms to emerge prior to pituitary findings, depressed mood, fatigue, and headaches occurred on average at 13 years, 12 years, and 11 years, respectively.

In cases where objective findings were established, no patterns of hormonal laboratory findings were consistently reported in the papers, with regard to ACTH-Adrenal axis, TSH-thyroid functioning, GH functioning, and LSH/FH functioning. Decreased levels of GH and cortisol, however, were reported in almost all the papers. Radiological findings were discussed in 57% of articles and the majority of papers did not mention specific locations in the pituitary. Microadenomas were reported 48% of the time with 52% considered adenomas.

Our literature review found 51% of cases reported were patients with depressive-like symptoms predating pituitary findings, as opposed to only 15% of cases that noted symptoms of mood disturbances after diagnosis or onset of pituitary pathology. Similarly, psychiatric morbidity has been evaluated in adult populations with hypopituitarism and growth hormone deficiency compared to age- and sex-matched adults with a different metabolic disorder: diabetes mellitus. Results indicated 46% of the adults in the growth hormone deficiency group were identified as psychiatric cases compared to only 24% of the adult patients with diabetes mellitus [4].

## 4. Conclusion

Pituitary adenomas often present with neuropsychiatric symptoms such as mood disorders. These early symptoms of pathology can be present even in the absence of objective findings of pituitary pathology. Patients can present with symptoms of depression and neurological symptoms prior to a diagnosis of underlying pituitary pathology. Our analysis suggests potential opportunities for early screening and diagnosis of pituitary adenoma. Further studies are needed to explore the use of depressive symptoms as early indicators of underlying pituitary pathology.

## Figures and Tables

**Table 1:** 

Hormone	Patient Level	Reference Range for Postmenopausal Female
Follicle Stimulating Hormone (FSH)	2.0 mIU/mL	40-250 mIU/mL

Luteinizing Hormone (LH)	1.3 mIU/mL	30-200 mIU/mL

ACTH	6.2 pg/mL	10-50 pg/mL

Morning Cortisol	1.8 ug/dL	5-23 ug/dL

Evening Cortisol	1.2 ug/dL	3-10ug/dL

Fasting GH	2.7 ng/mL	<5 ng/mL

**Table 2:** Review of selected literature on cases of patients presenting with hypopituitarism.

Article Title	Gender	Age	Patient Presentation	Type of Depression (Atypical or Typical)	Laboratory Findings (i.e. ACTH, Cortisol levels, Dexamethasone Test)	Radiologic Findings
Posttraumatic Panhypopituitarism with Depression [10]	M	38	Features of MDD, irritability, decreased libido, nausea, headache, cold intolerance, constipation, malaise, arthralgia, somnolence, and reduced psychomotor activity	N/A	LH 1.7 mlU/mL, FSH 6.2 mlU/mL, testosterone 0.01 nmol/L, basal cortisol 0.23 *μ*g/dL, PRL 0.28 ng/mL, free T3 1.3 pmol/L, free T4 1.1 pmol/L, TSH 0.06 mlU/L.	MRI: bilateral frontotemporal post traumatic encephalomalacia with gliosis and ex vacuo changes

Psychiatric morbidity in adults with hypopituitarism [11]	M, F	42.9 (mean)	GH deficiency, diabetes mellitus, major depression, and generalized anxiety disorder	41 Pt: MDD	GH deficiency	N/A

Sheehan's Syndrome Presenting as Major Depressive Disorder [12]	F	45	Generalized weakness, easy fatigability, loss of appetite, generalized body aches & pains and malaise. PMH included MDD and hypothyroidism	Atypical depression	Normocytic, normochromic anemia; cortisol 3.17 ug/dL, TSH 3.12 mIU/ml, FSH 3.00 mIU/l, LH 0.42 mIU/l, PRL 0.86 ng/ml, GH 0.22 ng/ml	MRI: empty sella

Personality in patients with pituitary adenomas is characterized by increased anxiety-related traits: comparison of 70 acromegalic patients with patients with nonfunctioning pituitary adenomas and age- and gender-matched controls [13]	M, F	45-70	Group 1: neurotic, harm avoidant, reduced novelty seeking behavior, especially lower impulsiveness, and high social conformity Group 2: neurotic and harm avoidant	N/A	Group 1: pituitary adenomas with acromegaly Group 2: nonfunctioning pituitary adenomas	Group 1: global enlargement of the grey matter

The impact of treatment on HPA axis activity in unipolar major depression [14]	M, F	31-57 (mean 46.33)	Unipolar major depression	1049 Pts: MDD, atypical depression and melancholic features	No changes in cortisol and ACTH levels before and after the treatment with antidepressants (56% of the patients)	N/A

Neuropsychiatric Manifestations in a Patient with Panhypopituitarism [15]	M	68	Agitation and aggressive behavior, disheveled, grossly disorganized speech & behavior, tangential thought process, lacked associational quality, delusions of paranoia & grandiosity, and rife with religious themes	Schizophrenia	CBC, kidney, liver function tests and urine toxicology within normal limits	MRI: prominent ventricles, subarachnoid spaces suggest gross atrophy, opacification of the left sphenoid sinus, transsphenoidal resection of the right lobe of the pituitary gland

Apathy and Pituitary Disease: It Has Nothing to Do with Depression [16]	Pt 1: M Pt 2: F Pt 3: F Pt 4: F	Pt 1: 48 Pt 2: 55 Pt 3: 47 Pt 4: 36	Pt 1: memory loss, concentration & attention problems Pt 2: memory problems, difficulty with expression, fatigue, depressed feelings, unmotivated, and intermittent suicidal thoughts Pt 3 & 4: lack of energy and motivation	Apathy syndrome	Pan-hypopituitarism after surgery to treat pituitary tumor	N/A

Increased adrenocorticotropic hormone levels predict severity of depression after six months of follow-up in patients in outpatients with major depressive disorder [17]	M, F	30-60	MDD	199 Pt: MDD	Patients with higher levels of ACTH at baseline were still depressed after treatment with SSRI, SNRI, and NaSSA	N/A

Atypical depression in growth hormone deficient adults, and the beneficial effects of growth hormone treatment on depression and quality of life [18]	16 M, 9 F	18-59 (mean 38.4)	Social isolation, decreased energy, sleep disturbances, pain, and mobility problems	25 Pt: typical or atypical depression	GH deficiency	N/A

Evidence for a differential role of HPA-axis function, inflammation and metabolic syndrome in melancholic versus atypical depression [19]	M, F	18-65	Melancholic features of depression	Atypical depression compared to melancholic depression	Melancholic depression shows hyperactivity of the Hypothalamic-Pituitary-Adrenal axis. Atypical depression is associated with hypofunctioning of the axis, inflammation and metabolic abnormalities	N/A

Biomarkers for Depression: Recent Insights, Current Challenges and Future Prospects [20]	M, F	N/S	MDD, treatment resistant depression, and atypical depression	MDD	Cortisol hyperactivity, overproduction of ACTH & CRH, and hypothyroidism. Inflammatory findings in depression including IL-6, IL-8; circadian rhythm changes	Reduced grey matter volume in hippocampal, prefrontal cortex, and basal ganglia regions

Depression and Hypothalamic-Pituitary-Adrenal Activation: A Quantitative Summary of Four Decades of Research [21]	M, F	18-75	Minor depression, anhedonia, psychotic depression	Atypical depression compared to nonatypical depression	Atypical depression shows lower levels of cortisol, ACTH, and CRH	Reduced grey matter volume in hippocampal, prefrontal cortex, and basal ganglia regions

Detection of Growth Hormone Deficiency in Adults with Chronic Traumatic Brain Injury [22]	M, F	41-43 (age at time of injury)	Memory and concentration impairments, decreased quality of life, anxiety, depression, social isolation, hyperlipidemia, weight gain, osteoporosis, and exercise intolerance	235 Pt: moderate depression	Hypopituitarism, especially GH deficiency and insufficiency, and testosterone deficiency	N/A

Cognitive effects of pituitary tumours and their treatments: two case studies and an investigation of 90 patients [23]	Pt 1: F Pt 2: F 90 Pt: M, F	Pt 1: 52 Pt 2: 63 90 Pt: 18-70	Pt 1: lethargic, easily fatigability, depressed mood, irritability, sleep and appetite disturbances Pt 2: hirsutism, mood change, cushingoid physical features, and memory loss	N/A	Pt 1: GH deficiency after radiation therapy to treat a pituitary adenoma Pt 2: Pan-hypopituitarism after trans-sphenoidal hypophysectomy to treat a pituitary adenoma	Pt 1: MRI- displacement of the optic chiasm, deformation of the third ventricle, and some lateral spread on the right side. Pt 2: MRI- no pathologies outside of the pituitary region

Neuropsychiatric Disturbances and Hypopituitarism after Traumatic Brain Injury in an Elderly Man [24]	M	77	Frontotemporoparietal subdural and subarachnoid hemorrhage after a traumatic brain injury. 2 months later, complained of headaches, dizziness, memory loss, visual and auditory hallucinations, and depressive symptoms. Symptoms improved with prednisone and levothyroxine	N/A	Pan-hypopituitarism.	N/A

Hypopituitarism as a consequence of traumatic brain injury (TBI) and its possible relation with cognitive disabilities and mental distress [25]	39 M, 28 F	38.8 (mean)	Patients with hormone deficiency presented with mild-moderate depression, anxiety, and psychoticism	8 Pt: severe depression 11 Pt: mild to moderate depression	GH deficiency (9% of patients) and Gonadotropin deficiency (9% of patients)	MRI: hypoxic-ischemic brain damage in neonatal brain injury PET scan: cortical asymmetry as well as hypometabolism

Pathophysiologic Aspects of Major Depression following Traumatic Brain Injury [26]	N/S	N/S	MDD, also including anxiety, substance use disorder, and unusual aggressive behavior	MDD and anxiety	GH deficiency, which was absent in the chronic stage of TBI and may have been associated with excessive fatigue, emotional disturbance, and lack of motivation	Major depression was associated with reduced gray matter volume in the lateral aspects of the left prefrontal cortex.

Chronic hypopituitarism after traumatic brain injury [27]	M, F	14-80 (mean 32)	Patients with major abnormal hormone deficiency had worse Disability Rating Scale score, depression, and quality of life in terms of energy, fatigue, emotional well-being, and general health	N/A	GH deficiency and insufficiency	CT: increased abnormal acute findings in patients with major hormonal deficiency

Complications after transsphenoidal surgery: our experience and a review of the literature [28]	N/S	N/S	Adenoma, acromegaly, Cushing's disease, prolactinoma, Rathke's cleft cyst, FSH secreting adenoma, granulomatous hypophysitis	Melancholic and atypical depression	Postoperative level of GH (<2 ng/l); postoperative level of serum cortisol (<50 nmol/l)	Postoperative CSF leak, thalamic infarct, hydrocephalus

Pituitary insufficiency after traumatic brain injury [29]	53 Pt (64.1% M)	45.2 ± 20.1 years, 45 (median)	Neuropsychological changes, like depression and anxiety, correlated more with the hemorrhagic lesions from brain injury compared to hypopituitarism	N/A	Cortisol, insulin-like growth factor 1, free thyroxine, estradiol, and testosterone were measured and showed pituitary insufficiency (25.4% of patients)	CT: skull fractures (61.5% of patients), one or more subarachnoid or intracerebral hemorrhagic lesions (73% of patients)

Hypopituitarism following brain injury: when does it occur and how best to test? [30]	N/S	N/S	Headache, irritability, loss of memory, attention deficit, depression, fatigue, low working capability, and cognitive changes	749 Pt: atypical depression	GH deficiency and low cortisol levels	MRI: hemorrhagic lesions

“M”: males; “F”: females; “Pt”: patient; “GH”: growth hormone; “FSH”: follicular stimulating hormone; “LH”: luteinizing hormone; “TSH”: thyroid stimulating hormone; “PRL”: prolactin; “ACTH”: adrenocorticotropic hormone; “SSRI”: selective serotonin reuptake inhibitor; “SNRI”: serotonin norepinephrine reuptake inhibitor; “NaSSA”: noradrenergic and specific serotonergic antidepressants; “TBI”: traumatic brain injury; “MDD”: Major Depression Disorder; “PTSD”: posttraumatic stress disorder; “MRI”: magnetic resonance imaging; “CT”: computed tomography; “PET”: positron emission tomography
